# Cobalt-Mediated Radical Copolymerization of Chlorotrifluoroethylene and Vinyl Acetate

**DOI:** 10.3390/polym11010101

**Published:** 2019-01-09

**Authors:** Pucheng Wang, Hu Wang, Qibao Dong, Ruke Bai

**Affiliations:** 1 Institute of Nuclear Physics and Chemistry, China Academy of Engineering Physics, Mianyang 621000, China; smilingwind@foxmail.com; 2 CAS Key Laboratory of Soft Matter Chemistry, Department of Polymer Science and Engineering, University of Science and Technology of China, Hefei 230026, China; wanghu@mail.ustc.edu.cn (H.W.); dongqb@mail.ustc.edu.cn (Q.D.)

**Keywords:** chlorotrifluoroethylene, fluorinated polymer, cobalt-mediated radical polymerization

## Abstract

Controlled radical copolymerization of chlorotrifluoroethylene (CTFE) and vinyl acetate (VAc) was successfully achieved in the presence of bis(acetylacetonato)cobalt(II) (Co(acac)_2_) as a mediated agent and 2,2′-azo-bis-isobutyronitrile (AIBN) as initiator. Both the molar mass and the fluorinated unit content of the copolymer could be controlled, and the chain extension polymerization of the obtained fluorinated copolymer was also achieved.

## 1. Introduction

Fluorinated polymers usually exhibit many remarkable properties due to the high electronegativity of the fluorine atom and the strong dissociation energy of the C-F bond (485 kJ·mol^−1^) [1,2,3,4,5]. However, perfluoropolymers often suffer from poor solubility in common organic solvents and have difficult processing characteristics. As a result, an increasing amount of research in recent years have been focusing on the copolymerization of fluoroolefins and non-fluoro monomers to prepare semi-fluorinated polymers [6,7,8,9,10,11,12,13,14,15,16,17,18,19,20,21,22,23,24,25]. Iodine transfer terpolymerization (ITP) was developed in the late 1970s by Tatemoto et al. [26] and believed to be a suitable controlled radical polymerization (CRP) method for the polymerization of some fluoroolefins, especially for vinylidene fluoride (VDF) [27,28,29]. However, there are very few successful cases publicly reported using this method. Since the mid-1990s, methods of CRP have been extensively studied and developed as a powerful tool for the synthesis of well-defined polymers with a predetermined molar mass, low dispersity, and various architectures [30,31,32,33,34]. Some of the CRP methods have also been used in the (co)polymerization of fluoroolefins, such as VDF, chlorotrifluoroethylene (CTFE), 3,3,3-trifluoropropene (TFP), and hexafluoropropylene (HFP). Ameduri et al. reported the synthesis of poly(VDF-co-TFP)-*b*-oligo(vinyl acetate) via the RAFT/MADIX process with fluorinated xanthate as a mediated agent [14]. Later on, another similar copolymerization process of perfluoro(methyl vinyl ether) and VDF was presented by Girard et al. [9]. In 2011, Liu et al. achieved the living/controlled radical copolymerization of CTFE and butyl vinyl ether (BVE) at room temperature under ^60^Co γ-ray irradiation with an *O*-ethyl xanthate [11]. Later in 2014, living/controlled radical copolymerization of CTFE and *N*-vinylpyrrolidone under ^60^Co γ-ray irradiation with S-benzyl *O*-ethyl dithiocarbonate as the RAFT/MADIX agent at room temperature was reported [6]. As mentioned before, VDF has been involved in many CRP reactions. Recently, Ameduri et al. reported another case in [35], where an organometallically mediated radical polymerization of VDF with an organocobalt compound R_0_(VAc)_n_Co^III^(acac)_2_ (R_0_ = primary radical generated by V-70, n ≈ 4) as initiator was achieved. Besides these, there were also some studies reporting the cobalt-mediated radical copolymerization of fluorinated monomers and VAc in the last two years [36,37,38]. However, the fluorinated monomers used in these works have no fluorine atom on the double bond, meaning that they have very similar chemical properties with non-fluorinated monomers.

**Scheme 1:**
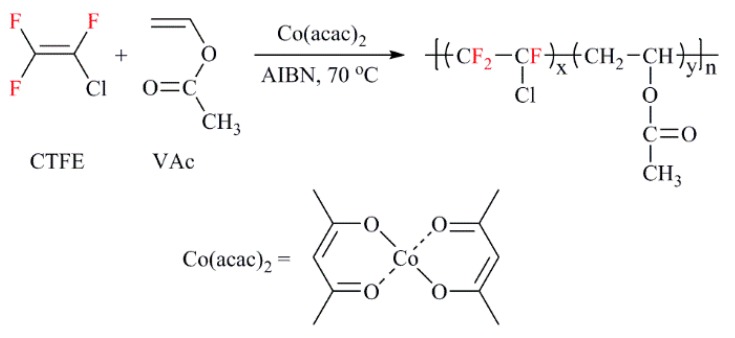
Schematic representation of Co(acac)_2_-mediated radical copolymerization of chlorotrifluoroethylene (CTFE) and vinyl acetate (VAc).

CTFE is a gas-state fluoroolefin, and still poses as an interesting challenge for the CRP of CTFE. In this work, we investigated the cobalt-mediated radical copolymerization of CTFE and VAc in the presence of bis(acetylacetonato)cobalt(II) (Co(acac)_2_) as a mediated compound. A more stable and easily available initiator, 2,2′-azo-bis-isobutyronitrile (AIBN), in replacement of V-70 was also used in the polymerization. The results indicate that the process has the features of CRP. Molar masses of the obtained copolymers increased linearly with the monomer conversions, and a linear relationship between ln([M]_0_/[M]) and the polymerization time was found to exist. Moreover, a block copolymer was prepared by chain extension polymerization of VAc using the obtained polymer as a macro-initiator and mediated agent. The Co(acac)_2_-mediated radical copolymerization process is depicted in Scheme 1.

## 2. Experimental

### 2.1. Materials

CTFE was purchased from Zhejiang Juhua Co., Ltd., Quzhou, China. VAc and ethyl acetate were purchased from Sinopharm Chemical Reagent Co, Ltd., Shanghai, China. VAc was purified by passing through a basic alumina column, and subsequently distilled. Ethyl acetate was refluxed and distilled over CaH_2_. AIBN and Co(acac)_2_ were purchased from Aladdin Industrial Corporation (Shanghai, China). AIBN was recrystallized from methanol twice before use. All other chemicals were used as received, unless otherwise noted.

### 2.2. Characterization

Weight percentages of carbon and hydrogen were assessed by elemental analysis on a Vario EL cube (Elementar, Langenselbold, Germany) instrument.

The values of the number-average molar mass (*M_n,GPC_*) and dispersity (*M_w_*/*M_n_*) were determined by means of a Waters 150 C gel permeation equipped with 10^3^, 10^4^, and 10^5^ Å Waters Ultrastyragel columns and a light scattering detector, using THF (1.0 mL/min) as the eluent, and the calibration was carried out with polystyrene standards.

^1^H and ^19^F NMR spectra were recorded on a Bruker DPX-400 spectrometer at room temperature, using CDCl_3_ as the solvent.

Thermogravimetric analysis (TGA) was performed with a DTG-60H (Shimadzu, Kyoto, Japan) apparatus, under nitrogen atmosphere, at a heating rate of 10 °C/min from room temperature up to a maximum of 700 °C.

### 2.3. Synthetic Procedures

#### 2.3.1. General Procedure for Co(acac)_2_-Mediated Copolymerization of CTFE and VAc

The reaction was performed in a 30 mL stainless steel autoclave equipped with a manometer, a safety inlet valve, and a magnetic stirrer. First, the mixture of 18 mg AIBN (0.11 mmol), 16 mg Co(acac)_2_ (0.055 mmol), 1.00 mL VAc (10.8 mmol), and 5.0 mL ethyl acetate were introduced into the autoclave, after which the vessel was closed and immersed in liquid nitrogen for 15 min. Several nitrogen-vacuum cycles were conducted to remove any traces of oxygen. After that, the required quantity (a certain molar fold of VAc (see in Table 1); 1 fold is 2.5 g) of CTFE was condensed into the autoclave via a mass flow meter. The exact mass value of CTFE was assessed by calculating the weight difference of the autoclave before and after filling with CTFE. Then, the polymerization reaction was conducted at 70 °C for a predetermined time. The reaction was quenched by cooling with ice water, and the residual CTFE was slowly vented. The solution was concentrated on a rotary evaporator and precipitated in petroleum ether. The obtained precipitate was finally dried under a vacuum at 40 °C until a constant weight was attained.

#### 2.3.2. General Procedure for Chain Extension Polymerization with VAc

The chain extension polymerization reaction was performed in the same autoclave we mentioned before. The obtained copolymer (0.215 g, Sample P7 in Table 1) and 1.00 mL (10.8 mmol) VAc was dissolved in 5 mL ethyl acetate and charged in the autoclave. Then, the vessel was closed and immersed in the liquid nitrogen for 15 min. Five cycles of nitrogen-vacuum were conducted to remove any traces of oxygen. After that, the autoclave was immersed in a thermostatic oil bath at 30 °C for 6 h. Then, the autoclave was cooled down by ice water and the solution was precipitated in petroleum ether. The resultant product was collected and dried under a vacuum at 40 °C until it reached a constant weight.

## 3. Results and Discussion

The results of Co(acac)_2_-mediated copolymerization of CTFE and VAc are shown in Table 1. In this work, AIBN was used to initiate the copolymerization instead of labile V70 at a higher temperature of 70 °C, which probably caused relatively higher values of dispersity of the resulted copolymers.

Equations (1) and (2) were used to determine the molar fractions of CTFE and VAc in the copolymer (CTFE% and VAc%, respectively) according to the molecular formulas of CTFE and VAc (CTFE = C_2_ClF_3_, VAc = C_4_H_6_O_2_):

C% = (2*M_C_* × CTFE% + 4*M_C_* × VAc%)/(*M_CTFE_* × CTFE% + *M_VAc_* × VAc%) (1)

CTFE% = 1 − VAc%(2)

where C% is the weight percentage of carbon in the copolymer accessed by elemental analysis. *M_C_* is the atomic weight of carbon. *M_CTFE_* and *M_VAc_* stand for the molar mass of CTFE and VAc, respectively.

The conversion of VAc (*Conv.*) could be calculated according to Equation (3):

(3) $$Conv.=\frac{m_{p}\times \frac{M_{VAc}\times \mathrm{VAc}\%}{M_{VAc}\times \mathrm{VAc}\%\;+\;M_{CTFE}\times \mathrm{CTFE}\%}}{m_{VAc}}$$

where *m_p_* is the mass of resultant copolymer, and *m_VAc_* is the initial mass of VAc in the solution.

The theoretical molar mass (*M_n,th_*) of poly(CTFE-co-VAc) was calculated according to Equation (4):

*M_n,th_* = 200 × *Conv.* × [*M_VAc_* + (CTFE%/VAc%) × *M_CTFE_*] (4)

where the coefficient 200 corresponds to the initial molar ratio of VAc to Co(acac)_2_.

Given that CTFE is a gas-state monomer, it is not a homogeneous reaction system for the copolymerization between CTFE and VAc. Only part of CTFE which we condensed in the autoclave dissolved in the reaction solution, and the undissolved CTFE would gradually permeate into the solution as the dissolved CTFE was consumed during the copolymerization reaction. There is no suitable method to determine the reactivity ratios for this kind of copolymerization system. Thus, we had to introduce some indirect parameters to describe the kinetics of the copolymerization.

**Figure 1:**
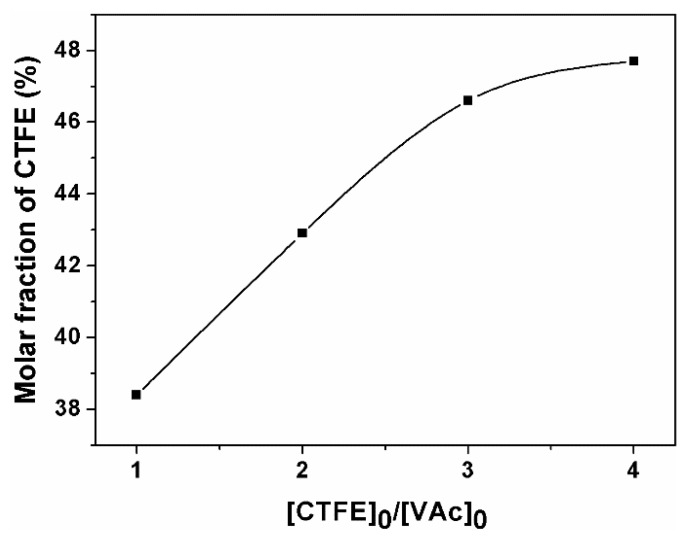
Molar content of CTFE in the copolymer as a function of the initial molar ratio of CTFE to VAc (Sample P1, P2, P3, and P4).

Figure 1 shows the molar content of CTFE in the obtained copolymers prepared under the same condition. As can be seen, the molar fractions of CTFE increase with the initial molar ratio of CTFE to VAc. Meanwhile, the rising trend comes to an equilibrium when [CTFE]_0_/[VAc]_0_ reaches up to 4. In the kinetic study to be described later, a four-fold initial molar ratio of CTFE to VAc was used in the polymerization process.

**Figure 2:**
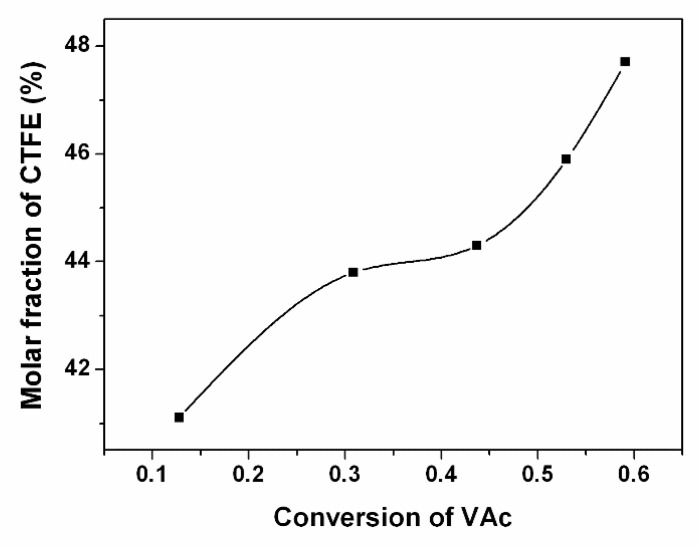
Molar content of CTFE in the copolymer as a function of VAc conversion (P4, P6, P7, P8, and P9).

When the initial molar ratio of CTFE to VAc is fixed at four-fold, molar fractions of CTFE in the resulted copolymers increase with the monomer conversions (shown in Figure 2), due to the increase in the real-time molar ratio of CTFE to VAc in the polymerization process.

**Figure 3:**
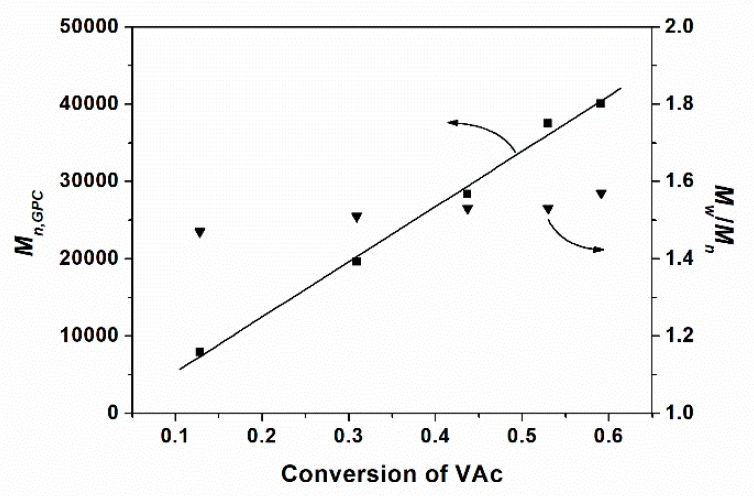
Molar mass and dispersity of the copolymer as a function of VAc conversion.

**Figure 4:**
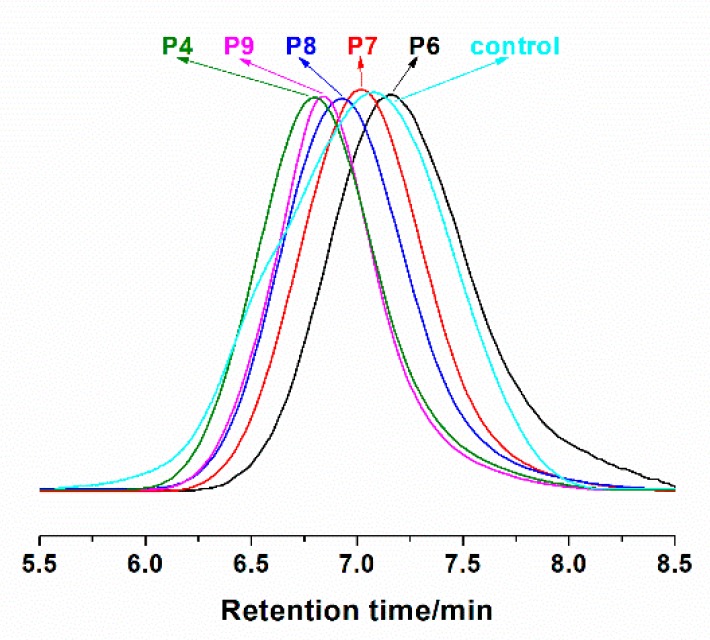
GPC traces of some representative copolymers.

It can be observed that molar masses determined by GPC (*M_n,GPC_*) are higher than the theoretical values (*M_n,th_*), which is probably due to the standards of polystyrenes used in the calibration. The molar masses of poly(CTFE-co-VAc) increase linearly with the monomer conversions, and the dispersities (*M_w_*/*M_n_*) remain at about 1.5 up to high conversions (shown in Figure 3). The linear kinetic curve could be ascribed to the quasi-alternating copolymerization of CTFE and VAc. Meanwhile, a control experiment with the same condition of sample P8 but without adding Co(acac)_2_ was conducted. The *M_n,GPC_* of the obtained copolymer is 15,500, and the value of dispersity is as high as 2.59. Figure 4 shows the GPC traces of these copolymers.

**Figure 5:**
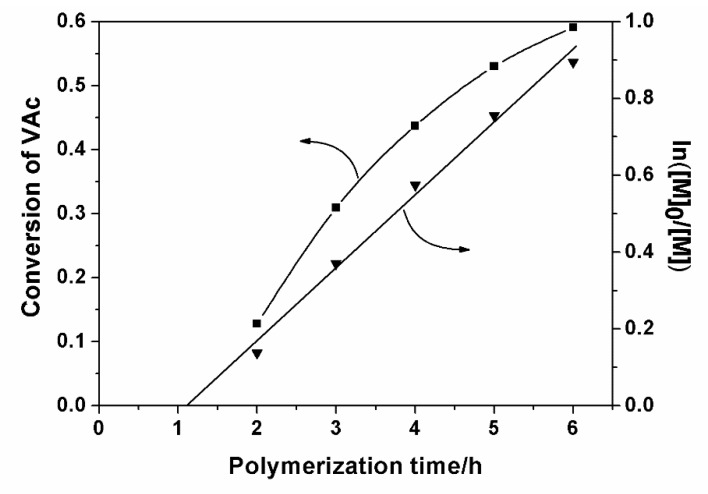
Conversion and ln([M]_0_/[M]) of VAc as a function of polymerization time for the copolymerization of CTFE and VAc.

Figure 5 shows the linear relationship between ln([M]_0_/[M]) and the polymerization time, which indicates that the Co(acac)_2_-mediated radical copolymerization of CTFE and VAc is a first-order reaction with respect to the monomer concentration. Meanwhile, an induction period of about one hour prior to polymerization was observed, which corresponds to the time required to inject a certain amount of radicals in the medium sufficient to convert the Co^II^ species into organocobalt(III) derivatives. In fact, the induction period of the copolymerization reaction in this work is much shorter than that previously reported for the polymerization of VAc by other researchers, which is as long as several hours [39,40]. This is probably due to the higher temperature and different chemical composition of the cobalt compound we used in the polymerization reaction. In fact, the exact denotation of the cobalt compound we used is Co(acac)_2_·2H_2_O, which means that there was at least a two-fold molar ratio of water to Co(acac)_2_ in the reaction medium. Water is a typical Lewis base, and would add to the coordination sphere of cobalt and facilitate the homolytic cleavage of the Co-C bond, which would open access to the reversible-termination mechanism from the very beginning of the process [41].

**Figure 6:**
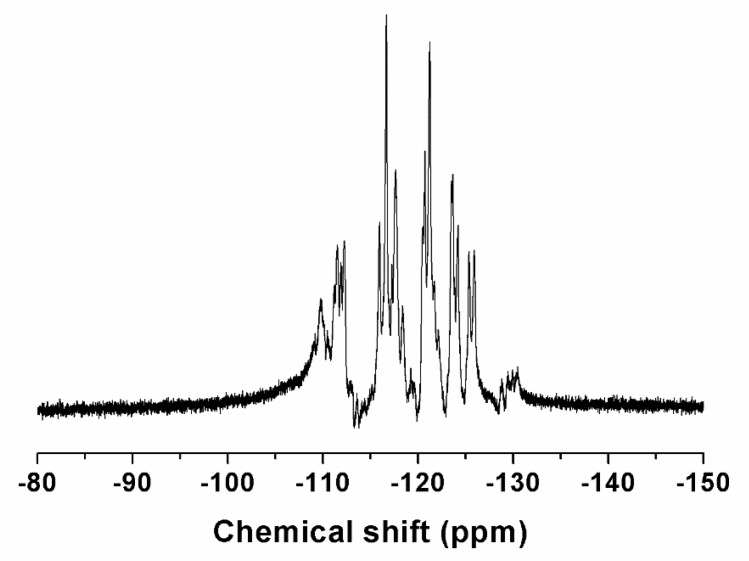
^19^F NMR spectrum of poly(CTFE-co-VAc) (Sample P7) recorded in CDCl_3_ at room temperature.

**Figure 7:**
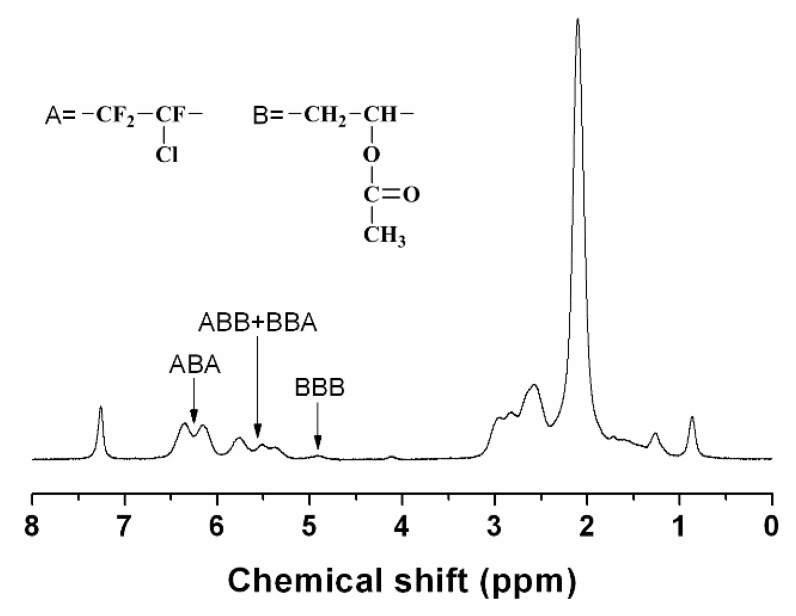
^1^H NMR spectrum of poly(CTFE-co-VAc) (Sample P7) recorded in CDCl_3_ at room temperature.

The chemical structure of the fluorinated copolymer was characterized by ^1^H and ^19^F NMR spectroscopy, and Sample P7 was chosen as a representative. For Sample P7, the molar ratio of the CTFE unit to VAc unit in the copolymer is 43.8/56.2, which is very close to the alternating structure. Figure 6 shows the ^19^F NMR spectrum of sample P7. There are a series of multiplets located between −108 and −132 ppm, which are attributed to the groups of C**F_2_** and C**F**Cl in the chain backbone. Meanwhile, no signal appears at −100 ppm in ^19^F NMR spectrum, which indicates that there are no CTFE-CTFE diads in the polymer chains [42]. Figure 7 shows the ^1^H NMR spectrum of poly(CTFE-co-VAc), where A represents the CTFE unit and B represents the VAc unit. The resonances located between 6.60 and 4.70 ppm can be attributed to the proton of methine in the VAc unit. As can be seen, there are four kinds of chain sequences in the copolymer, most of which have an alternating structure (A**B**A) that is followed by A**B**B or B**B**A, and very few B**B**B chain sequences exist. Signals appearing between 3.10 and 0.80 ppm belong to the methene of the VAc unit in the backbone and the methyl of the VAc unit in the pendent group.

**Figure 8:**
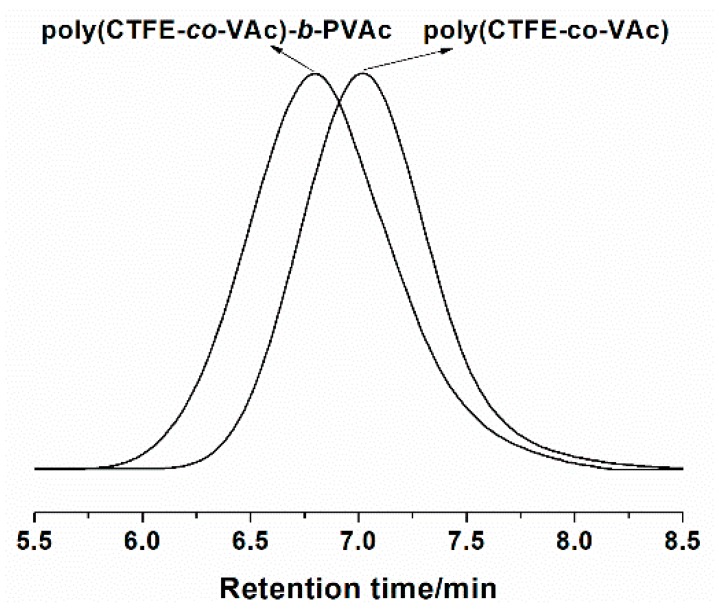
GPC traces of poly(CTFE-co-VAc) (Sample P7, *M_n,GPC_* = 19,600, *M_w_*/*M_n_* = 1.51) and poly(CTFE-co-VAc)-*b*-PVAc (*M_n,GPC_* = 39,300, *M_w_*/*M_n_* = 1.57).

Chain extension polymerization was conducted with Sample P7 as a macro-initiator and mediated agent, and VAc as the monomer. A really low reaction temperature of 30 °C avoided the initiating reaction of possible residual AIBN. Figure 8 shows the GPC traces of Sample P7 and the block copolymer (poly(CTFE-co-VAc)-*b*-PVAc) prepared by chain extension polymerization. As can be seen, the GPC curve of the block copolymer shifts toward a significantly higher molar mass compared with that of Sample P7, which indicates that the chain extension polymerization has been successfully achieved.

**Figure 9:**
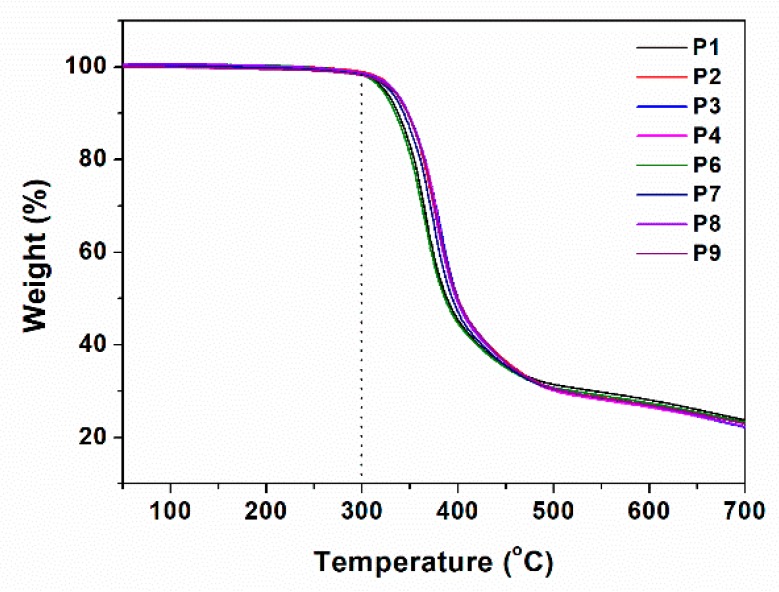
Thermogravimetric analysis (TGA) thermograms (under nitrogen, at 10 °C·min^−1^) of the copolymers.

Thermal stability of the obtained fluorinated copolymers was examined by thermogravimetric analysis (TGA) under a nitrogen condition. Figure 9 shows the thermograms of the copolymers. Severe degradation starts from about 300 °C, which demonstrates that the obtained fluorinated copolymers have good thermal stability.

## 4. Conclusions

We successfully conducted the radical copolymerization of CTFE and VAc in the presence of Co(acac)_2_ as a controlling agent. Molar fractions of the CTFE unit in the obtained copolymer gradually increased with the initial molar ratios of CTFE to VAc and the monomer conversions. The molar masses determined by GPC of the copolymers increased linearly with the VAc conversions. Moreover, a linear relationship between ln([M]_0_/[M]) and the polymerization time was found to exist. Besides, a block copolymer was prepared by chain extension polymerization of VAc based on the obtained fluorinated copolymer without adding any other extra agents. All these results demonstrated that Co(acac)_2_-mediated controlled radical copolymerization of CTFE and VAc initiated by AIBN could be achieved. Furthermore, we believe that this method would also be suitable for other electron-deficient fluoroolefins, and more detailed work needs to be done. VAc units in the polymer backbone could be transferred into hydroxyl groups, so that we could prepare various functional fluorinated polymers based on the method introduced in this work.

## Figures and Tables

**Table 1:** Results of Co(acac)_2_-mediated radical copolymerization of CTFE and VAc.

	Time (h)	[CTFE]_0_/[VAc]_0_ *^a^*	Elemental Analysis (wt%)		Copolymer Composition (mol%)		*Conv. ^b^*	*M_n,th_*	*M_n,GPC_*	*Ð ^d^*
			C	H	CTFE	VAc				
P1	6	1	39.66	3.57	38.4	61.6	0.586	18,600	36,300	1.53
P2	6	2	38.02	3.82	42.9	57.1	0.582	20,200	39,700	1.52
P3	6	3	36.73	3.75	46.6	53.4	0.587	22,000	40,400	1.52
P4	6	4	36.33	3.20	47.7	52.3	0.591	22,700	40,100	1.57
P5	1	4	-	-	-	-	N *^c^*	-	-	-
P6	2	4	38.68	3.84	41.1	58.9	0.128	4300	7900	1.47
P7	3	4	37.73	3.40	43.8	56.2	0.309	10,900	19,600	1.51
P8	4	4	37.55	3.08	44.3	55.7	0.437	15,600	28,400	1.53
P9	5	4	36.98	3.26	45.9	54.1	0.530	19,600	37,500	1.53

*^a.^* Initial molar ratio of CTFE to VAc. *^b.^* Conversion of VAc determined by gravimetry and elemental analysis. *^c.^* No product was obtained. *^d.^ Ð* = *M_w_*/*M_n_*.
